# Measuring objective physical activity in people with chronic low back pain using accelerometers: a scoping review

**DOI:** 10.3389/fspor.2023.1236143

**Published:** 2023-11-01

**Authors:** Mathilde Berger, Anne Martine Bertrand, Thomas Robert, Laurence Chèze

**Affiliations:** ^1^ Occupational Therapy Department (HETSL | HES-SO), University of Applied Sciences and Arts Western Switzerland, Lausanne, Switzerland; ^2^ Université de Lyon, Université Claude Bernard Lyon 1, Univ Eiffel, LBMC UMR_T 9406, Lyon, France

**Keywords:** physical activity, chronic low back pain, accelerometer, inertial measurement unit, scoping review

## Abstract

**Purpose:**

Accelerometers can be used to objectively measure physical activity. They could be offered to people with chronic low back pain (CLBP) who are encouraged to maintain an active lifestyle. The aim of this study was to examine the use of accelerometers in studies of people with CLBP and to synthesize the main results regarding the measurement of objective physical activity.

**Methods:**

A scoping review was conducted following Arksey and O'Malley's framework. Relevant studies were collected from 4 electronic databases (PubMed, Embase, CINHAL, Web of Science) between January 2000 and July 2023. Two reviewers independently screened all studies and extracted data.

**Results:**

40 publications out of 810 citations were included for analysis. The use of accelerometers in people with CLBP differed across studies; the duration of measurement, physical activity outcomes and models varied, and several limitations of accelerometry were reported. The main results of objective physical activity measures varied and were sometimes contradictory. Thus, they question the validity of measurement methods and provide the opportunity to discuss the objective physical activity of people with CLBP.

**Conclusions:**

Accelerometers have the potential to monitor physical performance in people with CLBP; however, important technical limitations must be overcome.

## 1. Introduction

Chronic low back pain (CLBP) has been defined as pain in the lumbar region persisting for more than 3 months (1). It is a major public health problem because of its high prevalence and its significant impact on the physical performance, psychological resources, and quality of life of affected individuals. It is a common cause of activity limitations, work disability and sick leave worldwide (2).

People with CLBP report high levels of disability and low levels of daily physical activity (3). They have difficulty maintaining their usual lifestyle habits, and their ability to perform work and leisure activities (4, 5). They describe their physical activity levels as reduced (6) and appear to have a high level of sedentary behavior. Sedentary behavior is defined as behavior adopted during low energy expenditure activities performed in a resting position like sitting or lying (6). Reducing sedentary behavior is a common treatment goal for people with CLBP (7). Several reasons for the reduction in physical activity levels have already been identified. One of these is the fear of experiencing pain (8). According to the fear-avoidance model, people with CLBP may interpret their pain as threatening and avoid exercise because of fear of reinjury (9). This can lead to physical deconditioning, disuse, depression, and disability (8). Other causes of activity limitation may include physical barriers such as pain intensity and comorbidities; psychological barriers such as lack of motivation, false beliefs, lack of perceived benefits, and misinterpretation; and socio-environmental barriers such as lack of time, work occupation, incorrect advice from health professionals and the family environment (10).

Nowadays, promoting physical activity and reducing sedentary behavior is widely recommended for people with CLBP. The management of this condition has progressed over the last 50 years from a biomedical to a biopsychosocial approach. Physical rest used to be systematically prescribed, whereas now the importance of physical activity and therapeutic education sessions is recognized (11). The World Health Organization defines physical activity as “*any bodily movement produced by skeletal muscles that requires energy expenditure*” (12). Daily physical activity, also known as free-living physical activity, refers to all movements regularly performed during leisure, employment, chores, sports and mobility activities (12). It is described according to several characteristics: intensity, duration, frequency, context and purpose (13). It can be measured using subjective or objective methods.

Assessments of physical activity are generally based on self-report measurements collected using personal records, activity diaries or questionnaires (14). The Baecke Physical Activity Questionnaire and the International Physical Activity Questionnaire are 2 examples of self-report measurements (14). These tools are inexpensive, easy to implement, and they measure individuals' perceptions of their own physical activity level (15). However, responses may be subject to recall and social desirability biases (15). For example, respondents may evaluate their level of physical activity differently from one session to another, or they may overestimate it.

The objective assessment of physical activity using motion sensors has progressed greatly over the last 2 decades. These sensors measure the biomechanical characteristics and/or physiological effects of physical activity (16). Initially developed in research laboratories, their use in the natural environment has been made possible by important technical advances related to the miniaturization of the tools (and consequently, the reduction of their weight and production costs), the optimization of battery autonomy, improvements in connectivity, and the optimization of data processing methods (17). Currently, accelerometers and inertial measurement systems (composed of an accelerometer, a gyroscope and possibly a magnetometer) are the gold standard for the measurement of activity in people with chronic diseases (18–21). They can be integrated into treatment methods and/or used to assess treatments effects.

However, little information is available about the use of motion sensors in people with CLBP. There are few studies in this population. Systematic reviews on the topic are outdated and include different methods of physical activity assessment (14, 22). Furthermore, data on the objective physical activity of people with CLBP are scarce and rarely compared to the objective physical activity of healthy people. Beyond the interest of producing objective research data, exploration of the different uses of accelerometers would allow for a more informed discussion of their potential benefits specifically within CLBP. Given the heterogeneity of studies in this specific population, a scope of the literature is required to inform researchers and clinicians about their use.

Thus, the main aims of this study were to examine the use of accelerometers in studies of people with CLBP and to synthesize the main results regarding the measurement of objective physical activity. More precisely, the first specific aim of this study was to map the use of accelerometers in studies of people with CLBP according to the models and number of sensors used, their position on the body, the duration of measurement, outcomes measured, and limitations. The second specific aim of this study was to synthesize the main findings for objective physical activity measured by accelerometers.

## 2. Methods

A scoping review was undertaken in accordance with the 5-stage methodological frameworks described by Arksey and O'Malley (23) and Levac et al. (24) as well as the PRISMA-ScR guidelines (Supplementary Table 1) (25).

### 2.1. Identification of the research questions

We defined 2 research questions. (1) How is objective physical activity measured using accelerometers in research involving people with CLBP? (2) What are the results of accelerometry studies of objective physical activity in people with CLBP?

### 2.2. Identification of relevant resources

Relevant articles were sought in 4 electronic databases (PubMed, Embase, CINHAL, Web of Science), and the reference lists of selected articles and conference proceedings were also searched. A complementary search was conducted in Google and Google Scholar to identify other scientific sources. Four categories of keywords (chronic low back pain, activity, accelerometer, evaluation) were defined by the 4 co-authors. Boolean search terms were used to combine subject keywords and synonyms. The search was performed in all fields of the databases screened and is described in Table 1. The search was limited to articles published between January 1, 2000 and July 1, 2023, due to the timeframe of clinical use of these instruments in healthcare and the rapid evolution of sensor technology.

**Table 1:** Keywords and search strategy.

Combiners	Terms
Chronic low back pain	(“Chronic low^a^ back pain” OR “low back pain” OR “back pain”) (all fields)
	AND
Physical activity	(Activity OR “physical activity” OR “functional activity” OR “daily activity” OR “everyday activity” OR “functional capacity” OR “physical performance”) (all fields)
	AND
Motion sensor	(Sensor OR “motion sensor” OR acceleromet^a^ OR actigraph^a^ OR “wearable tracker” OR “inertial measurement unit”) (all fields)
	AND
Evaluation	(Measure^a^ OR evaluat^a^ OR “objective^a^ measure^a^”) (all fields)
	NOT surgery (all fields)

^a^ Truncation symbols were used to allow for word variation searching.

### 2.3. Article selection

Inclusion criteria were defined prior to article selection. Exclusion criteria were specified during article selection. They were applied to all articles identified by the search.

Inclusion criteria:

• Study focusing on the assessment of people with CLBP (lumbar pain persisting beyond 3 months).
• Study focusing on the use of accelerometers as instruments to measure objective physical activity.
• Study focusing on daily physical activity data collection, or free-living data collection, over a period of several consecutive days.
• Article written in English or French.

Exclusion criteria:

• Study focusing on the evaluation of surgical techniques.
• Study focusing on the quantification of sleep duration and quality.
• Study focusing on specific populations (e.g., children, people over 75 years old or pregnant women).
• Study focusing on movement analysis or specifically analyzing muscle function.

First, 2 reviewers independently selected the articles according to titles, keywords and abstracts. Then, further selection was performed by the same 2 reviewers, based on the full-text review. Discrepancies were discussed and resolved with the help of 2 other reviewers with different backgrounds (research engineer and movement specialist).

### 2.4. Data charting

Data were extracted using a specifically created form. In addition to the usual information (authors, title, year of publication, and source) and study characteristics (objectives, study design, sample size and population), the following data were extracted: accelerometer models used, number of accelerometers used per person, position of accelerometers on the body, measurement duration, objective physical activity outcomes measured using the accelerometers, limitations of accelerometer use, main outcomes for objective physical activity.

### 2.5. Data analyses

First, a table was drawn to present the characteristics of the studies selected. The data were then examined and organized into the characteristics of use of accelerometers and objective physical activity outcomes. The characteristics of use were further analyzed and classified according to 6 themes defined by the 4 authors during the development of the reporting chart: accelerometer models, number of accelerometers used per person, placement of accelerometers, duration of measurement, outcomes, and limitations relating to accelerometer use. The occurrence and frequency of each characteristic identified were calculated.

The results of the studies were analyzed and classed into 6 subcategories consistent with the purpose of this review:

• Comparing the objective physical activity of participants with CLBP and asymptomatic participants.
• Comparing subjective physical activity (as estimated by self-questionnaires) and objective physical activity in both participants with CLBP and control participants.
• Comparing the objective physical activity of participants with CLBP with usual expert recommendations.
• Assessing factors associated with objective physical activity.
• Assessing the measurement properties of physical activity assessment scales or objective physical activity assessment techniques.
• Assessing changes in objective physical activity in participants with CLBP after a rehabilitation intervention.

Any disagreement in the categorization was discussed between the co-authors until agreement was reached.

## 3. Results

**Figure 1:**
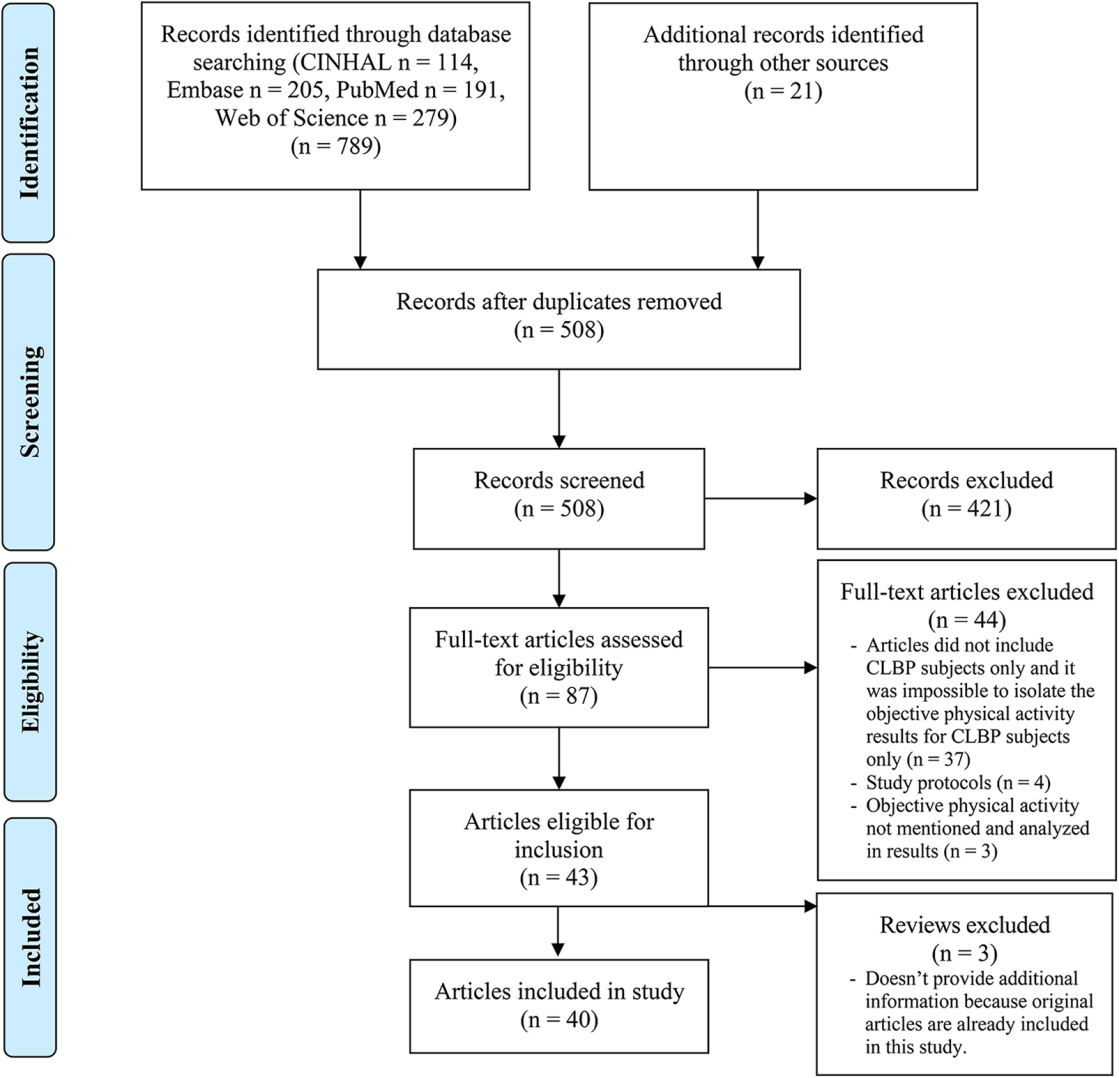
PRISMA flow chart.

A total of 810 articles were identified. The selection process is described in the PRISMA flow chart (Figure 1) (26). In total, 40 articles were selected for analysis (27–66). An overview of the articles included can be found in the supplementary data (Supplementary Table 2).

### 3.1. Use of accelerometers in people with CLBP

The use of accelerometers in people with CLBP is described in Table 2. A variety of accelerometer models were used to measure objective physical activity in people with CLBP. The triaxial Actigraph was the most often used in the included studies (*n* = 14, 36.9%). Two other models were also used many times: the ActivPAL (uniaxial or triaxial accelerometer) (*n* = 6, 15.4%) and the Fitbit (*n* = 3, 7.7%) (triaxial accelerometer, also known as an activity tracker).

**Table 2:** Description of the use of accelerometers in people with CLBP.

Themes	Description (source)	Number of studies^a^	% of studies^b^
Accelerometer model or device	Actigraph (27, 28, 31–33, 36, 42, 43, 49, 51, 52, 56, 59, 66)	14	36.9
	ActivPAL (35, 48, 50, 53–55)	6	15.4
	Fitbit (29, 41, 65)	3	7.7
	RT3 (44, 45)	2	5.1
	MT9 inertial 3D motion sensor (61, 62)	2	5.1
	BAN (sensor Mt-x and PDA) (38, 39)	2	5.1
	Actiwatch (27, 28)	2	5.1
	Micro Motion Logger Actigraph (46, 47)	2	5.1
	Dynaport ADL (58)	1	2.6
	PAMSys (34)	1	2.6
	GC data Concept (60)	1	2.6
	Tracmor (63)	1	2.6
	Lifecorder GS (57)	1	2.6
	Uniaxial accelerometer—model not specified (37, 64)	2	5.1
Number of accelerometers used per person	1 accelerometer (All except below)	35	89.7
	2 accelerometers (29, 35, 46, 51)	4	10.3
	3 accelerometers (58)	1	2.6
Position of accelerometers	Hip (30–33, 36, 38, 39, 43, 46, 49, 52, 56, 59, 62, 66)	15	38.5
	Right hip (30–33, 36, 38, 39, 43, 49, 52, 56, 59, 66)	13	33.3
	Lower back/waist/pelvis (37, 42, 51, 57, 58, 61, 63, 64)	8	20.5
	Wrist (27–29, 41, 46, 47, 65)	7	17.9
	Non-dominant wrist (27, 28, 46, 47)	4	10.3
	Thigh (35, 48, 50, 53, 54, 60)	6	15.4
	Sternum (34, 35)	2	5.1
	Left leg (58)	1	2.6
	Not specified (44, 45, 55)	3	7.7
Duration of measurement	7 days (29–33, 36, 37, 43, 46–51, 53–56, 59, 61, 64, 66)	22	56.4
	During waking hours (30, 31, 33, 36, 37, 43, 49, 51, 56, 59, 61, 64, 66)	13	33.3
	Day and night (46, 47, 50, 53, 54)	5	12.8
	2 weeks (38, 39, 42, 44, 45, 63)	6	15.4
	During waking hours (38, 39, 44, 45, 63)	5	12.8
	5 days (27, 28, 62)	3	7.7
	During waking hours (62)	1	2.6
	12 weeks (41, 57, 65)	3	7.7
	4–7 days (during waking hours) (52, 60)	2	5.1
	6 weeks (41)	1	2.6
	4 weeks (35)	1	2.6
	2 days (34)	1	2.6
	24 h (58)	1	2.6
Outcomes	Activity counts^c^ (27, 28, 30, 32, 33, 36–39, 42–47, 49, 51, 52, 56, 59, 61–64)	24	61.5
	Number of steps over time (29, 31–33, 41, 43, 48–50, 53–55, 57, 58, 60)	15	38.4
	Activity intensity level: sedentary, light, moderate, vigorous physical activity (29, 31, 36, 41, 43, 49, 51–53, 56, 59, 66)	12	30.8
	Body position: sitting, standing, walking, lying down (31, 34, 35, 48, 58, 60)	6	15.4
	Duration or number of sit-to-stand, stand-to-sit transitions (34, 53, 54, 60)	4	10.3
	Energy expenditure (MET) (35, 65, 66)	3	7.7
	Time spent in moderate to vigorous physical activity (32, 33)	2	5.1
	Walking parameters: duration, cadence, number of episodes, step frequency during walking (34, 48)	2	5.1
	Time standing, walking (50, 54)	2	5.1
	Motor activity (kcal) (57)	1	2.6
	Physical activity level (overall level that combines static or dynamic activity, trunk movement intensity, step frequency and trunk movement intensity) (58)	1	2.6
Limitations on the use of accelerometers	Lack of usable data (28, 29, 31–33, 35, 36, 38, 39, 42–45, 48, 51–53, 55, 56, 61, 65, 66)	22	56.4
	Wearing protocol not respected (29, 31–33, 36, 42, 43, 48, 51–53, 66)	12	30.8
	Technical failures of the instrument (38, 39, 44, 45, 51–53, 61, 65)	9	23.1

^a^ Data from 39 studies were finally considered for the analysis, the 40th study (40) used data from two other studies already included in the review (36, 51).

^b^ Total percentage per theme may be greater than 100% because some studies used more than one accelerometer model and measured more than one physical activity variable.

^c^ “Activity count” is a measure of activity intensity obtained by post-processing accelerometer data. However, the methodological details of how these “counts” are obtained are often specific to each device and are very rarely published.

Accelerometers were positioned on many different parts of the body to measure activity in people with CLBP. The most common position was the hip (*n* = 15, 38.5%), particularly the right hip (*n* = 13, 33.3%); however, they could be positioned on the lower back (*n* = 8, 20.5%), wrist (*n* = 7, 17.9%) or thigh (*n* = 6, 15.4%), and were occasionally positioned on the sternum (*n* = 5, 5.1%). In more than half of the studies included, the duration of physical activity measurement was 7 days (*n* = 22, 56.4%).

The objective physical activity outcomes measured in people with CLBP differed between studies. The average activity per minute, called “counts” (*n* = 25, 64.1%) and the number of steps (*n* = 15, 38.4%) were often reported. Around 30% of the studies reported time spent at different intensities of activity (sedentary, light, moderate, vigorous) (*n* = 12, 30.8%) whereas more than 15% focused on the postural components of physical activity (time spent sitting, standing, walking, lying, and number of transfers) (*n* = 6, 15.4%).

The use of accelerometers was limited by a lack of compliance with wearing the instrument for several days (*n* = 12, 30.8%), and technical failures (*n* = 9, 23.1%). More than half of the selected studies excluded participants because of a lack of usable data (*n* = 22, 56.4%).

### 3.2. Main objective physical activity outcomes in people with CLBP

The results of the studies are presented in Tables 3–8. Tables 3–5 synthesize the results of studies that reported the objective physical activity of people with CLBP measured with accelerometers. Specifically, Table 3 synthesizes the results of studies that reported the objective physical activity of people with CLBP compared to asymptomatic participants. The objective physical activity of people with CLBP appeared to differ from that of asymptomatic people. These differences were related to specific objective measures of physical activity. For example, people with CLBP spent more time in sedentary activities and less time in light-intensity activities during the day (59). Their level of objective physical activity differed from that of asymptomatic participants based on different times of the day (58, 61). They spent more time lying down and sitting in the evening (61). However, the total amount of objective physical activity of people with CLBP over a week did not seem to differ from that of asymptomatic people (60, 61).

**Table 3:** Main results comparing the objective physical activity of participants with CLBP and asymptomatic participants.

	(Source) Main results synthesized and reformulated
At least one difference in objective physical activity identified	(46) During the day, the mean number of active movements was significantly higher at the wrist than at the waist in both the CLBP (*n* = 20) and control groups (*n* = 20). The ratio of the amount of movement measured at the waist relative to that at the wrist was significantly lower in the LBP group than in the control group. There was a strong correlation (*r* = 0.880–0.965) between the number of movements in 1-min and the total movement measured at the wrist and at the waist for both groups during daytime.
	(58) There were significant differences in objective physical activity variables (lying time and walking step frequency) between participants with CLBP (*n* = 38) and non-symptomatic controls (*n* = 10) during the day but not at night. There were significant differences in objective physical activity variables (standing time, lying time, physical activity levels and walking step frequency) between participants with CLBP (*n* = 38) and non-symptomatic controls (*n* = 10) during the evening. Participants with CLBP had significantly weaker activity patterns compared to non-symptomatic controls during the day, especially in the evening.
	(59) There were significant differences in objective physical activity variables (mean daily minutes of sedentary time and in very light activity) between participants with CLBP (*n* = 22) and healthy controls (*n* = 155). Participants with CLBP (*n* = 22) spend more time sedentary and less time in very light activity. There was no significant difference in other objective physical variables (mean daily minutes in moderate or vigorous activity) between participants with CLBP (*n* = 22) and healthy controls (*n* = 155).
	(61) There was no significant difference in an objective physical activity variable (counts) between a group of participants with CLBP (*n* = 29) and a control group (*n* = 20) over a week but there was a significant difference in an objective physical activity variable (counts) between the two groups according to the time of the day. As compared to weekdays, the activity level of participants with CLBP (*n* = 29) was significantly higher in the morning, and lower in the evening than that of the control group. The activity level of the control group (*n* = 20) was significantly higher in the morning and significantly lower in the evening during the weekend as compared to weekdays, whereas the activity level of participants with CLBP (*n* = 29) was comparable during weekends and weekdays. There was no significant difference in the mean activity level between the three occupational subgroups of participants with CLBP (*n* = 8 working, *n* = 6 housekeeping, *n* = 13 invalidity benefits/sick leave) but all subgroups (*n* = 27) showed the same kind of activity pattern during the day: high physical activity level in the morning and low physical activity level in the evening.
No difference in objective physical activity identified	(60) There was no significant difference in objective physical activity variable (step counts, walking bouts, transfers, cadence, duration resting, duration walking and duration standing) between participants with CLBP (*n* = 26) and healthy controls (*n* = 20). There was a trend towards more standing time and more sit-stand transfers for participants with CLBP (*n* = 26) than healthy controls (*n* = 20).

**Table 4:** Main results comparing the subjective physical activity and objective physical activity in both participants with CLBP and asymptomatic.

	(Source) Main results synthesized and reformulated
Difference identified between subjective and objective physical activity	(56) Participants with CLBP (*n* = 25) and healthy controls (*n* = 53) both underestimated sedentary time (measured with the Global Physical Activity Questionnaire and compared to an accelerometer). Participants with CLBP (*n* = 25) underestimated sedentary time more than healthy controls (*n* = 53). Participants with CLBP (*n* = 25) overestimated moderate physical activity but not healthy controls (*n* = 53). Participants with CLBP (*n* = 25) and healthy controls (*n* = 53) both overestimated vigorous physical activity. Influencing factors in the overestimation of moderate physical activity identified were self-reported workplace, leisure time and transportation physical activity (measured with Global Physical Activity Questionnaire) (*n* = 41). Influencing factors in the overestimation of vigorous physical activity identified were self-reported workplace and leisure time physical activity (measured with Global Physical Activity Questionnaire) (*n* = 42).
	(62) There was a weak correlation between objective and subjective physical activity (*r* = −0.27) in participants with CLBP (*n* = 32) whereas there was a strong correlation between objective and subjective physical activity (*r* = 0.66) of healthy controls (*n* = 30). More participants with CLBP underestimate their physical activity level (*n* = 8) rather than overestimate (*n* = 4). Participants with CLBP who underestimated their physical activity level (*n* = 8) tended to score lowest on the “increasing activity” part in a coping scale (Coping Strategies Questionnaire) whereas participants with CLBP who overestimated physical activity level (*n* = 4) tend to have the lowest V_O2max_ score.

**Table 5:** Main results comparing the objective physical activity of participants with CLBP with usual expert recommendations.

(Source) Main results synthesized and reformulated
(52) Participants with CLBP (*n* = 46) spent 6% of their time per day in objective moderate to vigorous physical activity. A small proportion of participants (21.7%) achieved the recommended level of objective moderate to vigorous physical activity proposed by the WHO in 2010 (perform throughout the week a minimum of 150 min of moderate intensity aerobic physical activity (MPA), or at least 75 min of vigorous-intensity aerobic physical activity (VPA), or an equivalent combination of moderate- and vigorous-intensity activity (MVPA) in bouts of at least 10 min). A greater proportion of participants (84.8%) achieved the recommended level of objective moderate to vigorous physical activity proposed by the WHO in 2020 (perform throughout the week at least 150–300 min of MPA; or at least 75–150 min of VPA; or an equivalent MVPA throughout the week not including bouts of at least 10 min). There were no clinical or demographic differences between the groups of participants with CLBP that met the WHO recommendations and those that did not.

**Table 6:** Main results of the evaluation of factors associated with objective physical activity in participants with CLBP.

	(Source) Main results synthesized and reformulated
At least one association identified between factors and objective physical activity	(27) The responses of patients’ relatives with regard to the pain they notice in the categories of punishment, solicitation, and distraction assessed with the West Haven-Yale Multidimensional Pain Inventory were associated to objective physical activity (counts per minute) in participants with CLBP (*n* = 20). However, pain and cognitive-behavioral variables were not significantly predictive of objective physical activity.
	(28) There were three significant predictors of low physical activity level in participants with CLBP (*n* = 20): higher levels of fear of movement measured with the Tampa Scale for Kinesiophobia, more solicitous spousal responses measured with the West Haven-Yale Multidimensional Pain Inventory, more sensitive pain measured with the pressure algometer.
	(47) There were significant correlations between objective physical activity variables (counts) and gender, body mass index, muscle mass in people with CLBP (*n* = 66).
	(54) There were significant differences in objective physical activity variables, specifically upright and standing time, between distressed group (*n* = 9) and no-distressed group (*n* = 28) of participants with CLBP. Conversely, there was no difference in walking time and step count between groups. Depressive symptoms measured with a questionnaire (the Modified Zung Depression Index) could significantly predict upright time in people with CLBP.
	(32) There were significantly weak associations (*r* = 0.20–0.22) between some objective physical activity variables (time spent in moderate-to-vigorous physical activity per day, number of 10-minute bouts of moderate-to-vigorous physical activity per day) and disability (measured with the Roland Morris Disability Questionnaire) in participants with CLBP (*n* = 119). There was no significant association between objective physical activity variables (time spent in moderate-to-vigorous physical activity per day, time spent in light physical activity per day, counts per minute, number of 10-minute bouts of moderate-to-vigorous physical activity per day, number of steps per day) and fear of movement (measured with the Tampa Scale of Kinesiophobia) nor pain (measured by the Numerical Pain Rating Scale) in participants with CLBP (*n* = 119).
	(36) There were significant associations between being sufficiently physically active (objective physical activity: time spent per week with counts >2,020/min) and a lower body mass index as well as the physical active domain “work, sports and non-sport leisure time” (measured with the Baecke Physical Activity Questionnaire) in people with CLBP (*n* = 171). There were significant associations between being sedentary (objective physical activity: time spent per week with counts <100/min), depression (measured with the Beck Depression Inventory) and the educational level (primary school incomplete) of people with CLBP (*n* = 171). There was no significant association between being sufficiently physically active or being sedentary and the duration of symptoms, pain intensity (measured with the Numerical Pain Rating), disability (measured with the Roland Morris Disability Questionnaire), depression (measured with the Beck Depression Inventory) and fear of movement (measure with the Tampa Scale of Kinesiophobia).
	(35) There was a significant association between variation in objective physical activity (more in-bed hours) and experience of flare-ups defined by a 2-point increase in pain rating (measured with the Numeric Pain Rating scale). There was a significant association between variation in objective physical activity (greater total number of waking hours spent sedentary, greater percentage of waking hours spent sleeping, sedentary, or upright) and experience of flare-ups as defined by the participant (measured with the question “Did you experience a flare of low back pain today?”). There was a significant association between variation in objective physical activity (greater total time spent standing, greater percentage of waking hours when the participant was slow walking with a cadence of up to 74 steps per minute) and experience of flare-ups as defined by the participant (measured with the question “Did you experience a flare of low back pain today?”).
	(40) Based on previous studies (36, 51), it was suggested, using an Isotemporal Substitution Modeling (ISM), that replacing 60 min of sedentary behavior by 60 min of vigorous physical activity by week is associated with pain reduction (measured with the Numeric Pain Rating scale) in participants with CLBP (*n* = 358). Replacing 60 min of light or moderate physical activity by 60 min of vigorous physical activity by week is also associated with pain reduction in participants with CLBP (*n* = 358).
	(66) There were significant differences between objective physical activity of two profiles of participants with CLBP (participants with high level of central sensitization, and participants with low level of central sensitization; *n* = 42) using an advanced unsupervised machine learning approach: Hidden semi-Markov model. Participants with low levels of central sensitization had a higher transition probability from rest, light physical activity, and moderate-vigorous physical activity states to the sedentary state. They had a signiﬁcantly shorter bout duration of the sedentary state. Participants with high levels of central sensitization had longer durations of active and inactive states and had higher transition probabilities between active states.
No association identified between factors and objective physical activity	(44) There was no significant association between an objective physical activity variable (counts per minute) and pain intensity or depression (measured with a Visual Analogue Scales and with the Beck Depression Inventory II) in participants with CLBP (*n* = 66). However, the discrepancy between a self-reported daily life activity level (measured with an electronic diary) and an objective physical activity variable (counts per minute) was significantly associated with depression (measured with the Beck Depression Inventory II), but not with pain intensity.
	(45) There was no significant difference in an objective physical activity variable (counts per minute) in four profiles of participants with CLBP (avoiders, persisters, mixed performers, functional performers; *n* = 79). Furthermore, there was no significant association between an objective physical activity variable (counts per minute) and pain (measured with the Visual Analogue Scale).
	(49) After physical therapy treatment, there was no correlation (*r* = 0.002–0.08) between the changes in objective physical activity variables (counts per minute, total steps per day, total time spent in moderate-to-vigorous physical activity per day, light physical activity per day) and the changes in disability (measured with the Roland Morris Disability Questionnaire and the Quebec Back Pain Disability Scale) in participants with CLBP (*n* = 106).
	(43) There were no significant associations between objective physical activity variables (counts per minute, time spent on light physical activity per day, time spent in moderate to vigorous physical activity per day, number of steps per day) and pain intensity (measured with the Numerical Rating Scale) or disability (measured with the Roland Morris Disability Questionnaire) in participants with CLBP (*n* = 179).
	(66) There was no significant difference in an objective physical activity variable (counts per minute) in two profiles of participants with CLBP (participants with high levels of central sensitization, and participants with low levels of central sensitization; *n* = 42) using a conventional cut-points approach.

**Table 7:** Main results of the evaluation of measurement properties of physical assessment scales or other objective physical activity assessment techniques.

	(Source) Main results
Physical activity questionnaires	(30) There was no correlation between the sedentary time measured with an accelerometer and the total score of the Self-Reported Sedentary Behavior questionnaire, as well as the subscores by domain of the questionnaire, in participants with CLBP (*n* = 75).
	(33) There were weak significant correlations (*r* = 0.25–0.37) between some of the objective physical activity variables (total time spent in moderate-to-vigorous physical activity, total steps per day, count per minute, vector magnitude counts per minute measured with an accelerometer) and the scores of two self-reported physical activity questionnaires (the International Physical Activity Questionnaire long version and the Baecke Physical Activity Questionnaire) in participants with CLBP (*n* = 73). The highest significant correlations (*r* = 0.27–0.37) were found between questionnaire results and the effective number of steps by day (measured with an accelerometer), and between the International Physical Activity Questionnaire and some objective physical activity variables (counts per minute, vector magnitude counts per minute measured with an accelerometer).
	(47) Only the domains of “lumbar spine dysfunction” and “social life dysfunction” of the Japanese Orthopaedic Association Back Pain Evaluation Questionnaire were significantly correlated (*r* = 0.327; *r* = 0.321) with an objective physical activity variable (counts) in people with CLBP (*n* = 66). Low back pain (measured with a Visual Analogue Scale) was significantly correlated (*r* = 0.246) with an objective physical activity variable (counts) in people with CLBP (*n* = 66). There was no correlation between the objective physical activity variables (counts) and other domains of the Japanese Orthopaedic Association Back Pain Evaluation Questionnaire, the total score of the Oswestry Disability Index and that of the Roland-Morris Disability Questionnaire.
	(53) There were significantly weak relationships between most of the objective physical activity variables (upright time, stand time, walk time, step count) and self-reported functional disability (measured by Roland Morris Disability Questionnaire) in participants with CLBP (*n* = 38). There were significantly weak relationships between some objective physical activity variables (upright. time, stand time, walk time, step count) and physical performing tests (sit-to stand test, 50-ft walk test, 5-minute walk test).
	(62) There was a weak correlation (*r* = 0.27) between an objective physical activity variable (counts) and the amount of physical activity (measured with the Baecke Physical Activity Questionnaire) in participants with CLBP (*n* = 32). However, there was a strong significant correlation (*r* = 0.66) between an objective physical activity variable (counts) and the amount of physical activity (Baecke Physical Activity Questionnaire) in the control group (*n* = 20).
	(60) There was no correlation between objective physical activity variables measured with an accelerometer and by an inertia measurement unit. There were moderate correlations (*r* = 0.53–0.60) between perceived physical functioning (measured with the Oswestry Low Back Pain Disability Questionnaire) and cadence (measured with an accelerometer) as well as with walking speed and bending angle in block stepping (measured with an inertia measurement unit).
Doubly labelled water technique	(63) There was a strong significant correlation (*r* = 0.72) between an objective physical activity variable (counts/day) measured with an accelerometer and the physical activity level measured with doubly labelled water technique in participants with CLBP (*n* = 12). There was no significant correlation between physical activity (measured with both the accelerometer and the doubly labelled water technique) and disability (measured with Roland Morris Disability Questionnaire) in participants with CLBP (*n* = 12). Furthermore, there was no significant correlation between physical activity and fear of movement (measured with the Tampa Scale for Kinesiophobia) nor pain intensity (measured with a Visual Analogue Scale).
Accelerometers	(49) Objective physical activity variables (counts per minute, total steps per day, total time spent in moderate-to-vigorous physical activity per day, light physical activity per day) were not or poorly responsive to change (effect size and standardized response mean <0.18) in participants with CLBP (*n* = 106).

**Table 8:** Main results of the evaluation of the change in objective physical activity in people with CLBP following rehabilitation interventions.

	(Source) Main results
Significant change on objective physical activity	(37) There was a significant increase in an objective physical activity variable (movement count) in participants with CLBP (*n* = 3) after an intervention based on an education session combined with graded exposure sessions. However, there was no significant increase in an objective physical activity variable (movement count) in participants with CLBP after an intervention based on a single education session (*n* = 6) or an education session combined with graded operant activity sessions (*n* = 3).
	(38) There was a significant decrease in an objective physical activity variable (counts per minute) after discouraging feedback whereas there was a significant increase in an objective physical activity variable (counts per minute) after encouraging feedback messages during two feedback weeks in participants with CLBP (*n* = 16).
	(64) There was a significant improvement in an objective physical activity variable (counts) in participants with CLBP (*n* = 6) after the exposure to an *in vivo* program at 8 and 12 weeks (*n* = 3) of the follow-up but not after a graded activity program (*n* = 3).
	(57) There was a significant improvement in objective physical activity variables (steps per day, motor activity per day in kcal) in participants with CLBP (*n* = 20) after a workplace intervention. There was a significant improvement in objective physical activity variables (steps per day, motor activity per day in kcal) of people with CLBP (*n* = 20) at 3 and 6 months from the baseline. In contrast, there was no significant change in objective physical activity variables (steps per day, motor activity per day in kcal) in a control group (*n* = 17).
No significant change on objective physical activity	(39) There was no significant trend towards a pattern of activity similar to that of a control group (*n* = 60), after a personalized activity-based feedback intervention of 15 days in participants with CLBP (*n* = 17).
	(29) There was no significant difference in objective physical activity variables (light physical activity, moderate-to-vigorous physical activity, average step count per week) between participants with CLBP (*n* = 31) at 6 months of follow-up after an intervention promoting physical activity (individually physical activity program supported by coaching and the use of an activity tracker) and those of a control group of participants (*n* = 24) who received advice to stay active.
	(31) There was no significant difference in objective physical activity variables (steps count per day, mean percentage of the day spent performing sedentary, light, moderate-to-vigorous physical activity each day, mean percentage of the day spent in standing, lying and sitting positions) of participants with CLBP (*n* = 14) at one week of follow-up after a single physical therapy session. More specifically, there was no significant reduction in steps per day and time spent in sedentary activities, no significant increase in light to moderate physical activity, no significant decrease in time spent daily sitting and standing, and only significant increase in time spent lying.
	(34) There was no significant difference in objective physical activity variables (sitting time, standing time, walking time, maximum walking duration, lying time, total steps, walking duration variability, episode cadence average) of participants with CLBP (*n* = 8) between baseline measurements and immediately after a paravertebral spinal block and at 1 month of follow-up after a paravertebral spinal block.
	(41) There was no significant difference in an objective physical activity variable (mean step count) of participants with CLBP wearing Fitbit (*n* = 9) or pedometer (*n* = 8) between baseline measurements and immediately after a 6-week physical activity and lifestyle program designed to promote self-management of back pain and at 1 month of follow-up. However, aerobic fitness improved in participants wearing Fitbit (*n* = 9) whereas it remains stable in participants wearing a pedometer (*n* = 8) at 1 month of follow-up.
	(42) There was no significant difference in an objective physical activity variable (counts per minute) of participants with CLBP between baseline measurements and after 8 weeks of a supervised Nordic walking program (*n* = 25) or 8 weeks unsupervised Nordic walking program (*n* = 29).
	(50) There was no significant difference in objective physical activity variables (steps per day, sitting posture, variation of sitting posture, lumbar repositioning of participants with CLBP (*n* = 26) between baseline measurements and immediately and at 12 months of follow-up after a cognitive functional therapy.
	(55) There was no significant difference in an objective physical activity variable (step count) between a group of participants with CLBP who received a 6-week exercise program combined with an educational program (*n* = 20) and a group of participants with CLBP who received an educational program alone (*n* = 18).
	(60) There was no significant pre-post difference in objective physical activity variables (steps, walking bouts, transfers, cadence, duration resting, duration walking and duration standing) of participants with CLBP (*n* = 26) who received an individualized treatment aimed to obtain an optimal physical functioning, to attenuate pain and complaints and to make daily living more comfortable.
	(51) There was no significant pre-post difference in an objective physical activity variable (counts per minutes) of participants with CLBP who received a supervised group exercise therapy, physical activity coaching sessions and physical activity electronic feedback delivered by an activity monitor (*n* = 80), and participants with CLBP who received a supervised group exercise therapy alone (*n* = 80).
	(65) There was no significant pre-post difference in objective physical activity (MET) of participants with CLBP (*n* = 30) who received a pain self-management intervention involving wearable activity tracker and nurse consultations over 12 weeks.

Table 4 synthesizes the results of studies that reported the subjective physical activity and objective physical activity in both people with CLBP and asymptomatic people. Both people with CLBP and asymptomatic people underestimated their sedentary time and overestimated their time spent in vigorous activity (56). However, people with CLBP underestimated their sedentary time more than healthy controls (56). The correlation between objective and subjective physical activity in people with CLBP appeared to be weak (62).

Table 5 synthesizes the results of the only study that reported the objective physical activity of people with CLBP compared to usual expert recommendations. The results suggested that a significant proportion (84.8%) of people with CLBP met the current WHO recommendations for moderate to vigorous physical activity (52).

Table 6 presents the results of studies that reported factors associated with objective physical activity in participants with CLBP. The results indicated that the amount of physical activity performed by people with CLBP was associated with specific psychosocial factors (27, 28), distress (54), gender, body mass index and muscle mass (36, 47). But it did not appear to be associated with a specific level of pain (32, 36, 44, 45, 67), kinesiophobia, disability (32, 36, 49), or depression (36, 44).

Table 7 synthesizes the results of studies that evaluated the measurement properties of physical assessment scales (such as questionnaires) or other objective physical activity assessment techniques (such as the doubly labelled water technique). Many of the included studies showed that the results of physical activity questionnaires were not, or were only weakly, correlated with the objective physical activity variables obtained using accelerometers in people with CLBP. This was the case for many questionnaires: the Self-Reported Sedentary Behavior (30), the International Physical Activity Questionnaire (33), the Baecke Physical Activity Questionnaire (33, 62), the Japanese Orthopaedic Association Back Pain Evaluation Questionnaire (47), the Roland Morris Disability Questionnaire (47, 53). However, there was a strong significant correlation between objective physical activity measured with accelerometers and objective physical activity measured with the doubly labelled water technique (63). Only one study has evaluated the psychometric properties of accelerometers for measuring the objective physical activity in people with CLBP (49). This study demonstrated an absence or poor responsiveness to change of an accelerometer (Actigraph GT3X-BT) to measure the variables of objective physical activity intensity and step per day (49).

Finally, more than a third of the studies included in this scoping review (15 out of 40 studies) used accelerometers to assess changes in objective physical activity in people with CLBP following a rehabilitation intervention. Table 8 synthesizes the main results of changes in objective physical activity after various interventions. Few significant changes were found despite the diversity of rehabilitation treatments proposed. In fact, only 4 of 15 interventions had significant effects on objective physical activity variables. This was the case for an education session combined with graded exposure sessions (37, 64), a workplace intervention based on physical activity (57) and messages encouraging or discouraging activity sent on a phone application over 2 weeks (38). Interventions that did not show significant effects on objective physical activity variables included a physical activity program supported by coaching and the use of an activity tracker (27, 51, 65), a physical therapy session (31), a paravertebral spinal block (34), a program based on self-management of low back pain (41), a Nordic walking program (42), a cognitive functional therapy program (50), a 6-week exercise program combined with an education program (54), and an individualized program based on quality of life improvement (60).

## 4. Discussion

The main aim of this scoping review was to examine the use of accelerometers in studies of people with CLBP and to synthesize the main results regarding the measurement of objective physical activity. The main research findings obtained with accelerometers were contradictory because they sometimes identified differences, associations, and significant effects of interventions on objective physical activity, but not always. These disparate results could be related to differences in the ways the accelerometers were used as well as differences in data processing methods.

### 4.1. Accelerometers and people with CLBP

Several models of accelerometers used in the studies were identified. The outcomes measured using these different accelerometers were not directly comparable (16) since the conversion of acceleration signals depends on the instrument models (68) and on the software used to process the data. The Actigraph model was the most prevalent in the included studies and it is also the most widely used in the medical and research communities at large (69).

The position of the accelerometers on the body was variable. Most earlier studies of physical activity attached the instruments around the waist and hips. These positions were chosen because of their proximity to the body center of gravity (70); however, the inability of the instrument to detect upper limb and upper body movements in this position can lead to underestimation of physical activity (71). In addition, the possible interference with clothing and potential discomfort led to the exploration of other positions on the body (72). More recent studies favored wrist-worn accelerometers because they are easier for users to accept. Strong correlations were identified between wrist and waist measurements in people with CLBP (46). However, identifying basic aspects of physical behavior, such as postures and types of activities performed, is difficult using wrist-worn accelerometers. They are more susceptible to random noise compared to sensor data from other positions (e.g., the hip) because of variations in movement and posture of the upper limb (73). Placement of the accelerometer on the thigh has several advantages. The accelerometer can easily be worn under clothing, and it can be used to measure specific activities such as sitting, standing, lying down, walking, running, climbing, and pedaling (70). Results from a recent narrative review indicate that accelerometers placed on the wrist, waist, thigh, ankle and foot are considered more comfortable and acceptable by users than those placed on the neck, chest, trunk, elbow, and fingers (74). Thus, the most commonly suggested positions for people with CLBP (hip, lower back, waist and wrist) appear to be relevant and should be recommended for future research.

The most common duration of physical activity measurement in the included studies was 7 days. This duration allows the influence of specific physical activities that might be a one-off to be filtered out (75). However, this duration appears to be too short to account for the influence of seasons, weather, or holidays on the measurement of objective physical activity. The measurement period and weather conditions were not discussed in the results of the selected studies. In some cases, the measurement could exceed several weeks, especially when the aim of the study was to evaluate the effects of monitoring physical activity with an accelerometer-based treatment (41, 76).

Several limitations in the use of accelerometers were identified in the studies included. In addition to forgetting to put on the instrument (77), technical failures, such as battery malfunction (72) or failure of the accelerometer to recharge (78), contributed to later abandonment at home. To improve compliance with wearing the instrument for several days, a daily telephone call (72), an activity diary to complete, or a list of activities to be checked off daily were suggested (48). A small number of studies included in this review used these strategies. For example, Xu et al. proposed regular follow-up by nurses (65). An activity diary was suggested by several teams to provide information on missing accelerometric data (27, 37, 52).

### 4.2. Physical activity performed by people with CLBP

Overall, the relationship between physical activity and chronic low back pain is not yet well understood. Studies using objective measures of physical activity do not support the widespread belief that people with CLBP are less active than people without CLBP (22, 60, 61, 79). Although the amount of physical activity performed by people with CLBP is often considered to be reduced (14), the findings of this review show that, rather than being reduced overall, the level of physical activity varies depending on the specific physical activity outcomes measured. People with CLBP have a lower step frequency and spend more time lying down than controls (58). They are more active in the morning and less active in the evening than controls. They spend more time being sedentary and less time performing very light activity than controls (59). Therefore, as suggested by Griffin et al. it seems that it is mainly the distribution of activities performed during the day by people with CLBP that differs from that of people without CLBP (22). In fact, objective physical activity measurements show that people with CLBP are significantly less active in the evening and significantly more active in the morning compared to asymptomatic people (22). All these results confirm the need to consider perceived physical activity and objective physical activity as independent and complementary outcomes (60, 80).

### 4.3. Physical activity measured objectively with accelerometers as an outcome in studies evaluating change after a rehabilitation interventions

Few studies evaluating change in physical activity following rehabilitation interventions in people with CLBP found significant differences on objective physical activity. These results are analogous to those published in 2018 in a systematic review of people with different musculoskeletal disorders that found no effect of physical activity interventions on objective physical activity (81).

These findings raise questions about the content of interventions intended to modify participants' behavior toward a more active lifestyle (82). They also question the validity of physical activity measurement techniques (27, 33, 46, 53, 60, 62), their sensitivity and responsiveness for this population (49). Furthermore, the methods used to analyze accelerometry data may not be appropriate for people with CLBP. Classical analysis methods stratify activity into several levels (sedentary, light, moderate and vigorous), based on the activity count. These levels were initially developed for the field of cardiovascular research, in which moderate to vigorous activity was primarily considered to reflect a person's physical condition (68). The levels do not include light intensity activity that reflects the impact of disorders on daily physical performance. They do not specifically address light physical activity, which is particularly impacted in CLBP (59, 68, 83). Moreover, the reliability and validity of the activity variables measured using accelerometers have mostly been examined in healthy individuals (17). Studies of the psychometric properties of accelerometry to measure activity variables are still scarce in chronic pain, particularly CLBP (22, 53, 75).

### 4.4. Models of chronic pain

Our results question the common assumption that people with CLBP are less physically active than people without CLBP. High levels of pain and fear avoidance beliefs do not appear to be associated with objective physical activity levels (84). These findings provide an opportunity to question the fear-avoidance model proposed by Vlayen and Layton that suggests that the fear of pain generates disuse or a decreased level of physical activity (8). The results are in line with the avoidance-endurance model that suggests that a subgroup of people with CLBP ignore their pain and persist with activity (85). This latter model could help to understand the diversity of the results found by this scoping review as well as the clinical implications of these findings and the need to adapt rehabilitation programs to the different profiles of people with CLBP (85).

An increasing number of studies are attempting to identify different subgroups of people with CLBP using a multifactorial approach partly based on usual physical activity levels (86, 87). Such objective assessments of physical activity using accelerometers could help clinicians to understand the different subgroups of individuals with CLBP and to develop and provide individualized rehabilitation programs. However, only 2 of the included studies considered subgroups and provided separate results for the objective physical activity profiles of the people assessed (45, 66). Huijnen et al., showed that there was no significant difference in an objective physical activity variable (counts per minute) in 4 profiles of participants with CLBP (avoiders, persisters, mixed performers and functional performers) (45). Zheng et al., showed that there was no significant difference in an objective physical activity variable (counts per minute) in 2 profiles of participants with CLBP (participants with high levels of central sensitization, and participants with low levels of central sensitization) using a conventional cut-points approach, but there were significant differences between the objective physical activity levels of 2 profiles of participants with CLBP (participants with high levels of central sensitization and participants with low levels of central sensitization) using the Hidden semi-Markov model (66). The difference in results between these studies was probably related to the data processing techniques used; 2 used the cut-points approach (45, 66) and the other used an unsupervised machine learning approach (66). Thus, future treatment perspectives should be considered according to technical advances in accelerometry, data processing strategies, the validity of physical activity measurement techniques, and the feasibility of use of these tools with people with CLBP.

### 4.5. Perspectives

The opportunities offered by accelerometers seem promising for monitoring daily physical activity in people with CLBP. Accelerometers could provide measures of individual physical performance, i.e., what a person does in his or her real environment (69), and provide objective data regarding the intensity and extent of physical activity (60, 88). They could be used to define personalized activity goals to enrich educational sessions and self-management approaches (3). Furthermore, combining accelerometry with digital applications displaying physical activity data in a graphic format would allow people to visualize their own progress (14, 89, 90). Such applications could also contribute to the development of an individualized treatment plan (91). They could promote adherence to physical activity, reduce sedentary lifestyles (92–94) and, in addition, could help healthcare professionals to provide more sustainable support to their patients (93, 94). These instruments have already been used with people with other chronic illnesses such as type 2 diabetes or chronic obstructive pulmonary disease with promising results in terms of the quality of the follow-up (20, 95).

However, further research is obviously needed to address the methodological and technical limitations of accelerometers identified in this review. Objective physical activity data collected using accelerometers should be considered as independent of physical activity data collected by self-report measures. Regarding the use of accelerometers, clinicians and researchers need to be attentive to the choices made for use with people with CLBP, particularly in free-living conditions. The model of accelerometer, the position on the body, and the duration of measurement should be determined with caution because of the many biases that can arise (related to the diversity of occupations and environments of the wearers). Several strategies need to be implemented to address issues of non-compliance and potential technical incidents (such as regular reminders, the use of a dairy, and a support service available every day). The processing of accelerometer data should be more transparent to facilitate data sharing between different research teams. This would allow better understanding of the explanatory models of the disease so that more specific treatments could be proposed.

### 4.6. Study strengths and limitations

To our knowledge, this is the first scoping review to map the use of accelerometers in people with CLBP. The results should help clinicians and researchers interested in the use of these instruments in the development of future projects. For example, to better define the physical activity of people with CLBP or to assess the effects of interventions more accurately in this group. Furthermore, the results provide an opportunity to increase our understanding of the objective physical activity of people with CLBP. However, this study has some limitations. Only 4 electronic databases were searched. All included articles were in English. The optional step proposed by Arksey and O'Malley's method of presenting the results to users was not performed. Discussion of the results with healthcare professionals and people with CLBP who have used accelerometers would have enriched our thinking. Furthermore, the objective physical activity data measured by accelerometers were impossible to compare due to the diversity of device models used. Although the results of physical activities were expressed in “counts” in over 60% of the studies, the detailed on the post-processing used to obtain these counts was device-dependent and rarely specified.

The design of the current study did not allow rigorous assessment of the methodological quality of the selected articles. The included studies about the objective physical activity of people with CLBP measured with accelerometers after rehabilitation interventions were mainly different types of quasi-experimental studies whose results should be confirmed with more rigorous methodological designs. As such, the results provided an overview of how accelerometers are used in studies of people with CLBP, but analysis of the potential effect of their use on physical activity in people with CLBP was not possible. The results mainly suggest hypotheses and perspectives for further studies.

## 5. Conclusion

To conclude, this scoping review mapped the use of accelerometers to objectively measure physical activity in people with CLBP. Although accelerometers have been used in several studies to this purpose, there is no consensus on their use within this specific population. The main results related to objective physical activity are sometimes contradictory. Although accelerometers may indeed prove to be particularly useful for monitoring physical activity in people with CLBP, further research is needed to resolve the identified technical and methodological limitations to confirm this potential.

## Data Availability

The raw data supporting the conclusions of this article will be made available by the authors, without undue reservation.
