# Supplementary material for: Measuring objective physical activity in people with chronic low back pain using accelerometers: a scoping review
Source: Front Sports Act Living. 2023 Nov 1;5:1236143. doi: 10.3389/fspor.2023.1236143 (PMC10646390; doi:10.3389/fspor.2023.1236143)
Supplement: Supplementary file 2 [file Table2.docx]

Supplementary data:

Appendix B. First author, year, journal, design study, accelerometer model used, sample size and sub-category classification in included studies

| **Reference number** | **First author and year** | **Journal** | **Design study^a^** | **Accelerometer model used^b^** | **Sample size** | **Sub-category classification^c^** |
| --- | --- | --- | --- | --- | --- | --- |
| (27) | Alschuler *et al.*, 2011 | Clinical Journal of Pain | Unspecified | Actiwatch | n = 20 people with CLBP | 4 |
| (28) | Alschuler *et al.*, 2011 | Pain | Unspecified | Actiwatch | n = 20 people with CLBP | 4 |
| (29) | Amorim *et al.*, 2019 | BMC Musculoskeletal Disorders | Pilot randomized controlled trial | ActiGraph GT3X+ (triaxial accelerometer) + Fitbit | n = 68 people with CLBP | 6 |
| (30) | Araujo *et al.*, 2020 | PM & R: the journal of injury, function, and rehabilitation | Cross-sectional study | ActiGraph GT3X-BT (triaxial accelerometer) | n = 75 people with CLBP | 5 |
| (31) | Brewer *et al.*, 2017 | BMC Research Notes | Pilot study | ActiGraph GT3XP-BTLE (triaxial accelerometer) | n = 14 people with CLBP | 6 |
| (32) | Carvalho *et al.*, 2017 | Archives of Physical Medicine and Rehabilitation | Cross-sectional study | ActiGraph wGT3X-BT (triaxial accelerometer) | n = 119 people with CLBP | 4 |
| (33) | Carvalho *et al.*, 2017 | Musculoskeletal Science and Practice | Cross-sectional study | ActiGraph (triaxial accelerometer) | n = 73 people with CLBP | 5 |
| (34) | Chuan Yen *et al.*, 2018 | Sensors | Unspecified | PAMSys (triaxial accelerometer) | n = 8 people with CLBP | 6 |
| (35) | Costa *et al.*, 2022 | Pain | Longitudinal case-crossover study | ActivPAL3 (triaxial accelerometer)*2 | n = 126 people with CLBP | 4 |
| (36) | Damato *et al.*, 2021 | Journal of Manipulative and Physiological Therapeutics | Cross-sectional study | ActiGraph GT3X (triaxial accelerometer) | n = 171 people with CLBP | 4 |
| (37) | de Jong *et al.*, 2005 | Clinical Journal of Pain | Single case experimental design | Unspecified (uniaxial accelerometer) | n = 6 people with CLBP | 6 |
| (38) | Dekker-van Weering *et al.*, 2012 | Applied Psychophysiology and Biofeedback | Prognostic cohort study | Body Area Network (Mt-x sensor and Personal Digital Assistant) (triaxial accelerometer) | n = 16 people with CLBP | 6 |
| (39) | Dekker-van Weering *et al.*, 2015 | Disability and Rehabilitation | Prognostic cohort study | Body Area Network (Mt-x sensor and Personal Digital Assistant) (triaxial accelerometer) | n = 17 people with CLBP | 6 |
| (40) | Freire *et al.*, 2022 | Archives of Physical Medicine and Rehabilitation | Cross-sectional study | ActiGraph GT3X (triaxial accelerometer) | n = 358 people with CLBP | 4 |
| (41) | Gordon *et al.*, 2017 | The Journal of Sports Medicine and Physical Fitness | Unspecified | Fitbit Charge HR (triaxial accelerometer) | n = 17 people with CLBP | 6 |
| (42) | Hartvigsen *et al.*, 2010 | BMC Musculoskeletal Disorders | Single blind randomized clinical trial | Actigraph GT 265 | n = 136 people with CLBP | 6 |
| (43) | Hisamatsu *et al.*, 2022 | Journal of Physical Activity and Health | Prospective cohort study | ActiGraph GT3X-BT(triaxial accelerometer) | n = 179 people with CLBP | 4 |
| (44) | Huijnen *et al.*, 2010 | Pain | Unspecified | RT3 (triaxial accelerometer) | n = 66 people with CLBP | 4 |
| (45) | Huijnen *et al.*, 2011 | European Journal of Pain | Unspecified | RT3 (triaxial accelerometer | n = 79 people with CLBP | 4 |
| (46) | Inoue *et al.*, 2019 | Spine Surgery and Related Research | Validation study | Micro Motion Logger Actigraph (omnidirectional accelerometer)*2 | n = 20 people with CLBP ;  n = 20 people without CLBP | 1 |
| (47) | Inoue *et al.*, 2019 | European Spine Journal | Unspecified | Actigraph Micro-Motion logger (omnidirectional accelerometer) | n = 66 people with CLBP | 4, 5 |
| (48) | McDonough *et al.*, 2013 | Clinical Journal of Pain | Feasibility randomized controlled trial | ActivPAL (uniaxial accelerometer) | n = 57 people with CLBP |  |
| (49) | Morelhão *et al.*, 2018 | Clinical Rehabilitation | Prospective cohort study | ActiGraph GT3X-BT (triaxial accelerometer) | n = 106 people with CLBP | 4, 5 |
| (50) | O’Sullivan *et al.*, 2015 | Physical Therapy | Multiple case-cohort study | ActivPAL | n = 26 people with CLBP | 6 |
| (51) | Oliveira *et al.*, 2022 | Journal of Orthopaedic & Sports Physical Therapy | Randomized controlled trial | ActiGraph GT3X (triaxial accelerometer) + Fitbit | n = 160 people with CLBP | 6 |
| (52) | Reneman *et al.*, 2022 | Archives of Physical Medicine and Rehabilitation | Cross-sectional study | ActiGraph GT9X (triaxial accelerometer) | n = 46 people with CLBP | 3 |
| (53) | Ryan *et al.*, 2008 | Journal of Back & Musculoskeletal Rehabilitation | Unspecified | ActivPAL | n = 38 people with CLBP | 5 |
| (54) | Ryan *et al.*, 2010 | Manual Therapy | Cross-sectional study | ActivPAL (uniaxial accelerometer) | n = 38 people with CLBP | 4 |
| (55) | Ryan *et al.*, 2010 | Manual Therapy | Single-blind pilot randomized controlled trial | ActivPAL | n = 38 people with CLBP | 6 |
| (56) | Schaller *et al.*, 2016 | BioMed Research International | Cross-sectional study | ActiGraph GT3X+ (triaxial accelerometer) | n = 27 people with CLBP ;  n = 53 people without CLBP | 2 |
| (57) | Shimo *et al.*, 2021 | Journal of Back & Musculoskeletal Rehabilitation | Pilot randomized controlled study | Lifecorder | n = 37 people with CLBP | 6 |
| (58) | Spenkelink *et al.*, 2002 | Clinical Rehabilitation | Unspecified | Dynaport ADL Monitor (3 uniaxial accelerometers) | n = 47 people with CLBP  n = 10 people without CLBP | 1 |
| (59) | Tomkins-Lane *et al.*, 2022 | The Spine Journal | Cross-sectional study | ActiGraph | n = 22 people with CLBP ;  n = 155 people without CLBP | 1 |
| (60) | van Rooij *et al.*, 2015 | Disability and Rehabilitation | Unspecified | GC Dataconcepts | n = 26 people with CLBP ;  n = 20 people without CLBP | 1, 5, 6 |
| (61) | van Weering *et al.*, 2009 | European Journal of Pain | Cross-sectional study | MT9 inertial 3D motion sensor (3 uniaxial accelerometers) | n = 29 people with CLBP ;  n = 20 people without CLBP | 1 |
| (62) | van Weering *et al.*, 2011 | Clinical Rehabilitation | Cross-sectional study | MT9 inertial 3D motion sensor (3 uniaxial accelerometers) | n = 32 people with CLBP ;  n = 20 people without CLBP | 2, 5 |
| (63) | Verbunt *et al.*, 2001 | Archives of Physical Medicine and Rehabilitation | Case-control study in a cross-sectional design | Tracmor (triaxial accelerometer) | n = 13 people with CLBP ;  n = 13 people without CLBP | 5 |
| (64) | Vlaeyen *et al.*, 2002 | Clinical Journal of Pain | A replicated crossover single-case (with alterning treatments) design | Unspecified (uniaxial accelerometer) | n = 6 people with CLBP | 6 |
| (65) | Xu *et al.*, 2023 | BMC Nursing | Longitudinal study | Fitbit Flex 2 | n = 30 people with CLBP | 6 |
| (66) | Zheng *et al.*, 2023 | Computer Methods and Programs in Biomedicine | Unspecified | ActiGraph GT3X (triaxial accelerometer) | n = 42 people with CLBP | 4 |

^a^Study design as quoted by the authors
^b^Accelerometer model as quoted by the authors
^c^Subcategories as defined in the data analyses in which the objective physical activities results of the study were classified: (1) Comparing the objective physical activity of participants with CLBP and asymptomatic participants; (2) Comparing subjective physical activity (as estimated by self-questionnaires) and objective physical activity in both participants with CLBP and control participants ; (3) Comparing the objective physical activity of participants with CLBP with usual expert recommendations; (4) Assessing factors associated with objective physical activity; (5) Assessing the measurement properties of physical activity assessment scales or objective physical activity assessment techniques; (6) Assessing change in the objective physical activity of participants with CLBP following rehabilitation interventions.
